# Comparison of probabilistic Boolean network and dynamic Bayesian network approaches for inferring gene regulatory networks

**DOI:** 10.1186/1471-2105-8-S7-S13

**Published:** 2007-11-01

**Authors:** Peng Li, Chaoyang Zhang, Edward J Perkins, Ping Gong, Youping Deng

**Affiliations:** 1 School of Computing, University of Southern Mississippi, Hattiesburg, MS 39406, USA; 2 Environmental Laboratory, U.S. Army Engineer Research and Development Center, 3909 Halls Ferry Rd. Vicksburg, MS, 39180, USA; 3 SpecPro Inc., 3909 Halls Ferry Rd, Vicksburg, MS, 39180, USA; 4 Department of Biological Sciences, University of Southern Mississippi, Hattiesburg, MS 39406, USA

## Abstract

**Background:**

The regulation of gene expression is achieved through gene regulatory networks (GRNs) in which collections of genes interact with one another and other substances in a cell. In order to understand the underlying function of organisms, it is necessary to study the behavior of genes in a gene regulatory network context. Several computational approaches are available for modeling gene regulatory networks with different datasets. In order to optimize modeling of GRN, these approaches must be compared and evaluated in terms of accuracy and efficiency.

**Results:**

In this paper, two important computational approaches for modeling gene regulatory networks, probabilistic Boolean network methods and dynamic Bayesian network methods, are compared using a biological time-series dataset from the Drosophila Interaction Database to construct a Drosophila gene network. A subset of time points and gene samples from the whole dataset is used to evaluate the performance of these two approaches.

**Conclusion:**

The comparison indicates that both approaches had good performance in modeling the gene regulatory networks. The accuracy in terms of recall and precision can be improved if a smaller subset of genes is selected for inferring GRNs. The accuracy of both approaches is dependent upon the number of selected genes and time points of gene samples. In all tested cases, DBN identified more gene interactions and gave better recall than PBN.

## Background

The development of high-throughput genomic technologies (i.e., DNA microarrays), makes it possible to study dependencies and regulation among genes on a genome-wide scale. In last decade, the amount of gene expression data has increased rapidly necessitating development of computational methods and mathematical techniques to analyze the resulting massive data sets. In order to understand the functioning of cellular organisms, why complicated response patterns to stressors are observed, and provide a hypothesis for experimental verification, it is necessary to model gene regulatory networks (GRNs). Currently, clustering, classification and visualization methods are used for reconstruction or inference of gene regulatory networks from gene expression data sets. These methods generally group genes based on the similarity of expression patterns. Based on large-scale microarray data retrieved from biological experiments, many computational approaches have been proposed to reconstruct genetic regulatory networks, such as Boolean networks [1,2], differential equations [1,3], Bayesian networks [4-6] and neural networks [7]. Among these approaches, Boolean network methods and Bayesian network methods have drawn the most interest in the field of systems biology.

Much recent work has been done to reconstruct gene regulatory networks from expression data using Bayesian networks and dynamic Bayesian network (DBN). Bayesian network approaches have been used in modeling genetic regulatory networks because of its probabilistic nature. However, drawbacks of Bayesian network approaches include failure to capture temporal information and modeling of cyclic networks. DBN is better suited for characterizing time series gene expression data than the static version. Perrin et al. [8] used a stochastic machine learning method to model gene interactions and it was capable of handling missing variables. Zou et al. [9] presented a DBN-based approach, in which the number of potential regulators is limited to reduce search space. Yu et al. [10] developed a simulation approach to improve DBN inference algorithms, especially in the context of limited quantities of biological data. In [11], Xing and Wu proposed a higher order Markov DBN to model multiple time units in a delayed gene regulatory network. Recently, likelihood maximization algorithms such as the Expectation-Maximization (EM) algorithm have been used to infer hidden parameters and deal with missing data [12].

The Boolean Network model, originally introduced by Kauffman [1,13,14] is also very useful to infer gene regulatory networks because it can monitor the dynamic behaviour in complicated systems based on large amounts of gene expression data [15-17]. One of the main objectives of Boolean network models is to study the logical interactions of genes without knowing specific details [17,18]. In a Boolean network (BN), the target gene is predicted by other genes through a Boolean function. A probabilistic Boolean network (PBN), first introduced by Shmulevich et al. in [16,19] is the stochastic extension of Boolean network. It consists of a family of Boolean networks, each of which corresponds to a contextual condition determined by variables outside the model. As models of genetic regulatory networks, the PBN method has been further developed by several authors. In [20], a model for random gene perturbations was developed to derive an explicit formula for the transition probabilities in the new PBN model. In [21], intervention is treated via external control variables in a context-sensitive PBN by extending the results for instantaneously random PBN in several directions. Some learning approaches for PBN have also been explored [22-24]. Considering the same joint probability distribution over common variables, several fundamental relationships of two model classes (PBN and DBN) have been discussed in [25].

In this paper, two important computational approaches for modeling gene regulatory networks, PBN and DBN, are compared using a biological time-series dataset from the Drosophila Interaction Database [26] to construct a Drosophila gene network. We present the PBN and DBN approaches and GRN construction methods used and discuss the performance of the two approaches in constructing GRNs.

## Results

A real biological time series data set (Drosophila genes network from Drosophila Interaction Database) was used to compare PBN and DBN approaches for modeling gene regulatory networks [27,28]. The raw data was preprocessed in the same way as given in [29]. There were 4028 gene samples with 74 time points available in *Drosophila melanogaster *genes network through the four stages of the life cycle: embryonic, larval, pupal and adulthood [27]. An example network of drosophila muscle development is given in [29], in which muscle-specific protein 300 (*Msp*-300) is treated as hub gene in their inferred network. We used a different subset of the genes which participate in the development of muscle. Particularly, *Mlp*84B and other genes which contribute to larval somatic muscle development were used to infer gene regulatory networks.

The *D. melanogaster *gene Muscle LIM protein at 84B (abbreviated as *Mlp*84B) has also been known in FlyBase as Lim3. It encodes a product with putative protein binding involved in myogenesis which is a component of the cytoplasm. It is expressed in the embryo (larval somatic muscle, larval visceral muscle, muscle attachment site, pharyngeal muscle and two other listed tissues). Table 1 shows the scores of *Mlp*84B interacting with other related genes [26].

**Table 1:** The interactions and scores of Mlp84B with other genes

High Confidence	Scores	Other interactions	Scores
CG10722	0.5642	*Cdk*7	0.3569
CG13501	0.9005	*Impe*1	0.1108
CG17440	0.5811	*Pfk*	0.3155
CG7046	0.6626	*TfIIB*	0.2436
CG7447	0.5411	*Stck*	0.2523
CG11115 (*Ssl*1)	0.7917	*tup*	0.1094

**Figure 1:**
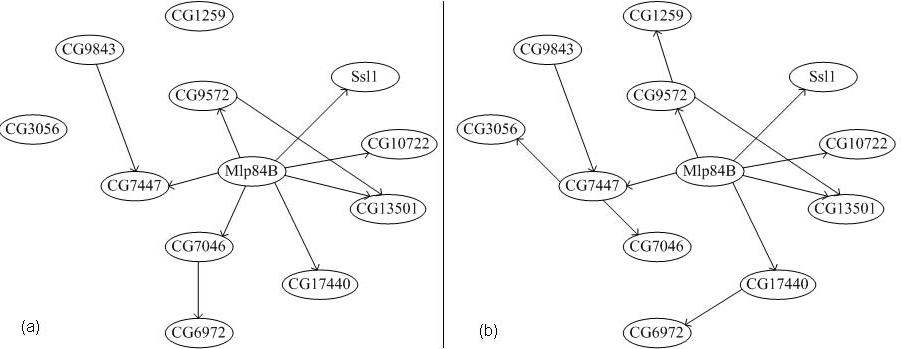
**Drosophila larval somatic muscle development network**. The genetic network inferred by PBN. (b)The genetic network inferred by DBN

Here, we first selected 12 genes to infer GRNs using PBN and DBN. The constructed GRNs are shown in Figure 1. There exists 18 interactions totally within this small larval somatic muscle network [26]. 10 and 12 interactions in the network have been successful identified. Most interactions between *Mlp*84B and genes with high confidence have been inferred.

More comparison results of PBN(n, e) and DBN(n, e) are given in Table 2, where n is the number of nodes (genes) in network and e the number of edges (interactions) among the nodes. PBN(30, 60) means that there are 30 nodes and 60 edges in that PBN simulation. To analyze the effect of network size on the inference accuracy, four (n, e) combinations, (12, 18), (20, 35), (30, 60) and (40, 80), were considered for inferring gene network. For each combination, we randomly selected five subsets of genes of the same numbers of genes and edges from the Drosophila gene network. For each subset genes, we inferred a gene network and retrieved the number of correct edges *Ce*, miss errors *Me*, and false alarm errors *Fe*. For each combination (n, e), the average and range of *Ce*, *Me *and *Fe *were calculated, as given in Table 2. A correct edge is the one that exists in a real network (i.e. the Drosophila gene network) and is successfully identified by the inference methods. Miss error is defined as the edge between two genes that exists in a real network, but the inference algorithms miss or make wrong orientations. False alarm error is the edge that the inference algorithms create but does not exist in the real network.

**Table 2:** Comparison of PBN and DBN methods using different sample networks

	Miss errors *Me*			False alarm errors *Fe*			Correct edges *Ce*			Accuracy (%) (*R*, *P*)		Time(s) *T*
	
	min	max	avg	min	max	avg	min	max	avg	recall	precision	avg
PBN(12,18)	2	9	6.4	0	4	2.4	6	9	7.8	54.9	76.5	13.2
PBN(20,35)	12	22	16.8	3	6	4.8	11	15	13.6	44.7	73.9	19.7
PBN(30,60)	33	41	36.0	7	10	8.0	17	20	18.4	33.8	69.6	27.9
PBN(40,80)	48	63	55.4	4	6	5.6	18	22	19.6	26.1	77.8	39.2
DBN(12,18)	3	8	5.8	1	3	2.2	9	11	10.4	64.2	82.5	20.1
DBN(20,35)	13	17	15.2	4	7	5.4	14	18	16.8	52.5	75.7	36.0
DBN(30,60)	30	39	33.6	11	15	12.6	24	30	20.2	37.5	61.6	50.6
DBN(40,80)	46	57	51.2	5	9	7.4	28	34	22.8	30.8	75.5	87.6

We used the benchmark measures recall *R *and precision *P *to evaluate performances of inference algorithms for PBN and DBN. While different definitions for recall and precision exist [30], in this paper, *R *is defined as *Ce*/(*Ce *+ *Me*) and *P *is represented as *Ce*/(*Ce *+ *Fe*). The selection of subset genes in network was based on the current existing gene interactions and network diagram in the Drosophila genes network [26].

The results in Table 2 show that for the same (n, e) case, DBN reduces miss errors but increases false alarms errors slightly. For all cases, DBN can identify more corrected edges than PBN and hence improve recall. The precision of DBN is better in three cases but worse in one case than PBN. For both PBN and DBN methods, recall and precision decrease if the number of genes increases. One can see that if more genes are selected for inferring GRNs, the network contains more edges and it is more difficult to successfully identify the interactions among genes. While the DBN method can give better recall of identifying genetic network interactions, it is more time-consuming than PBN.

## Discussion

It is challenging to infer GRNs from time series gene expression data. Among thousands of genes, each gene interacts with one or more other genes directly or indirectly through complex dynamic and nonlinear relationships, time series data used to infer genetic networks have low-sample size compared to the number of genes, and gene expression data may contain a substantial amount of noise. Different approaches may have different performances for different datasets. Moreover, inference accuracy depends not only upon models but also on inference schemes. In this paper, we only select two representative inference algorithms for PBN and DBN to model the GRNs, respectively. It is desirable to perform a more comprehensive evaluation of the two approaches with different inference methods and to develop the more robust algorithm and techniques to improve the accuracy of inferring GRNs.

## Conclusion

PBN-based and DBN-based methods were used for inferring GRNs from Drosophila time series dataset with 74 time points obtained from the Drosophila Interaction Database. The results showed that accuracy in terms of recall and precision can be improved if a smaller subset of genes is selected for inferring GRNs. Both PBN and DBN approaches had good performance in modeling the gene regulatory networks. In all tested cases, DBN identified more gene interactions and gave better recall than PBN. The accuracy of inferring GRNs was not only dependent upon the model selection but also relied on the particular inference algorithms that were selected for implementation. Different inference schemes may be applied to improve accuracy and performance.

## Methods

### Boolean network and probabilistic Boolean network

In a BN, the expression level of a target gene is functionally related to the expression states of other genes using logical rules, and the target gene is updated by other genes through a Boolean function. There are only two gene expression levels (states) in a Boolean network (BN): on and off, which are represented as "activated" and "inhibited". A probabilistic Boolean network (PBN) consists of a family of Boolean networks and incorporates rule-based dependencies between variables. In a PBN model, BNs are allowed to switch from one to another with certain probabilities during state transitions. Since PBN is more suitable for GRN reconstruction from time series data and a Boolean network is just a special case of PBN and we only consider PBN for comparison.

#### Boolean network

We use the same definition as in [2,18] for a Boolean network. A Boolean network *G*(*V*, *F*) is defined by a set of nodes (variables) representing genes *V *= {*x*_1_, *x*_2_,..., *x*_*n*_} (where *x*_*i *_∈ {0, 1} is a binary variable) and a set of Boolean functions *F *= {*f*_1_, *f*_2_,..., *f*_*n*_}, which represents the transitional relationships between different time points. A Boolean function f(xj1(i),xj2(i),...,xjk(i)(i))
 MathType@MTEF@5@5@+=feaafiart1ev1aaatCvAUfKttLearuWrP9MDH5MBPbIqV92AaeXatLxBI9gBaebbnrfifHhDYfgasaacH8akY=wiFfYdH8Gipec8Eeeu0xXdbba9frFj0=OqFfea0dXdd9vqai=hGuQ8kuc9pgc9s8qqaq=dirpe0xb9q8qiLsFr0=vr0=vr0dc8meaabaqaciaacaGaaeqabaqabeGadaaakeaacqWGMbGzcqGGOaakcqWG4baEdaWgaaWcbaGaemOAaO2aaSbaaWqaaiabigdaXaqabaWccqGGOaakcqWGPbqAcqGGPaqkaeqaaOGaeiilaWIaemiEaG3aaSbaaSqaaiabdQgaQnaaBaaameaacqaIYaGmaeqaaSGaeiikaGIaemyAaKMaeiykaKcabeaakiabcYcaSiabc6caUiabc6caUiabc6caUiabcYcaSiabdIha4naaBaaaleaacqWGQbGAdaWgaaadbaGaem4AaSMaeiikaGIaemyAaKMaeiykaKcabeaaliabcIcaOiabdMgaPjabcMcaPaqabaGccqGGPaqkaaa@4E40@ with *k*(*i*) specified input nodes is assigned to node *x*_*i*_. The gene status (state) at time point *t *+ 1 is determined by the values of some other genes at previous time point *t *using one Boolean function *f*_*i *_taken from a set of Boolean functions *F*. So we can define the transitions as

xi(t+1)=f(xj1(i)(t),xj2(i)(t),...,xjk(i)(i)(t))     (1)
 MathType@MTEF@5@5@+=feaafiart1ev1aaatCvAUfKttLearuWrP9MDH5MBPbIqV92AaeXatLxBI9gBaebbnrfifHhDYfgasaacH8akY=wiFfYdH8Gipec8Eeeu0xXdbba9frFj0=OqFfea0dXdd9vqai=hGuQ8kuc9pgc9s8qqaq=dirpe0xb9q8qiLsFr0=vr0=vr0dc8meaabaqaciaacaGaaeqabaqabeGadaaakeaacqWG4baEdaWgaaWcbaGaemyAaKgabeaakiabcIcaOiabdsha0jabgUcaRiabigdaXiabcMcaPiabg2da9iabdAgaMjabcIcaOiabdIha4naaBaaaleaacqWGQbGAdaWgaaadbaGaeGymaedabeaaliabcIcaOiabdMgaPjabcMcaPaqabaGccqGGOaakcqWG0baDcqGGPaqkcqGGSaalcqWG4baEdaWgaaWcbaGaemOAaO2aaSbaaWqaaiabikdaYaqabaWccqGGOaakcqWGPbqAcqGGPaqkaeqaaOGaeiikaGIaemiDaqNaeiykaKIaeiilaWIaeiOla4IaeiOla4IaeiOla4IaeiilaWIaemiEaG3aaSbaaSqaaiabdQgaQnaaBaaameaacqWGRbWAcqGGOaakcqWGPbqAcqGGPaqkaeqaaSGaeiikaGIaemyAaKMaeiykaKcabeaakiabcIcaOiabdsha0jabcMcaPiabcMcaPaaa@60AE@

where each *x*_*i *_represents the expression value of gene *i*, if *x*_*i *_= 0, gene *i *is inhibited; if *x*_*i *_= 1, it is activated. The variable *j*_*k*(*i*) _represents the mapping between gene networks at different time points. Boolean function *F *represents the rules of regulatory interactions between genes.

#### Probabilistic Boolean network

Probabilistic Boolean network inference is the extension of Boolean network methods to combine more than one possible transition Boolean functions, so that each one can be randomly selected to update the target gene based on the selection probability, which is proportional to the coefficient of determination (COD) of each Boolean function. Here we briefly give the same notation of PBN as in [19]. The same set of nodes *V *= {*x*_1_, *x*_2_,..., *x*_*n*_} as in a Boolean network is used in a PBN *G*(*V*, *F*), but the list of function sets *F *= {*f*_1_, *f*_2_,..., *f*_*n*_} is replaced by *F *= {*F*_1_, *F*_2_,... *F*_*n*_}, where each function set Fi={fj(i)}j=1,2,...l(i)
 MathType@MTEF@5@5@+=feaafiart1ev1aaatCvAUfKttLearuWrP9MDH5MBPbIqV92AaeXatLxBI9gBamXvP5wqSXMqHnxAJn0BKvguHDwzZbqegyvzYrwyUfgarqqtubsr4rNCHbGeaGqiA8vkIkVAFgIELiFeLkFeLk=iY=Hhbbf9v8qqaqFr0xc9pk0xbba9q8WqFfeaY=biLkVcLq=JHqVepeea0=as0db9vqpepesP0xe9Fve9Fve9GapdbaqaaeGacaGaaiaabeqaamqadiabaaGcbaGaemOray0aaSbaaSqaaiabdMgaPbqabaGccqGH9aqpcqGG7bWEcqWGMbGzdaqhaaWcbaGaemOAaOgabaGaeiikaGIaemyAaKMaeiykaKcaaOGaeiyFa03aaSbaaSqaaiabdQgaQjabg2da9iabigdaXiabcYcaSiabikdaYiabcYcaSiabc6caUiabc6caUiabc6caUiabdYgaSjabcIcaOiabdMgaPjabcMcaPaqabaaaaa@56D8@ composed of *l*(*i*) possible Boolean functions corresponds to each node *x*_*i*_. A realization of the PBN at a given time point is determined by a vector of Boolean functions. Each realization of the PBN maps one of the vector functions fk=(fk(1)(1),fk(2)(2),...fk(n)(n))
 MathType@MTEF@5@5@+=feaafiart1ev1aaatCvAUfKttLearuWrP9MDH5MBPbIqV92AaeXatLxBI9gBaebbnrfifHhDYfgasaacH8akY=wiFfYdH8Gipec8Eeeu0xXdbba9frFj0=OqFfea0dXdd9vqai=hGuQ8kuc9pgc9s8qqaq=dirpe0xb9q8qiLsFr0=vr0=vr0dc8meaabaqaciaacaGaaeqabaqabeGadaaakeaacqWGMbGzdaWgaaWcbaGaem4AaSgabeaakiabg2da9iabcIcaOiabdAgaMnaaDaaaleaacqWGRbWAcqGGOaakcqaIXaqmcqGGPaqkaeaacqGGOaakcqaIXaqmcqGGPaqkaaGccqGGSaalcqWGMbGzdaqhaaWcbaGaem4AaSMaeiikaGIaeGOmaiJaeiykaKcabaGaeiikaGIaeGOmaiJaeiykaKcaaOGaeiilaWIaeiOla4IaeiOla4IaeiOla4IaemOzay2aa0baaSqaaiabdUgaRjabcIcaOiabd6gaUjabcMcaPaqaaiabcIcaOiabd6gaUjabcMcaPaaakiabcMcaPaaa@5035@, 1 ≤ *k *≤ *N*, 1 ≤ *k*(*i*) ≤ *l*(*i*), where fk(i)(i)∈Fi
 MathType@MTEF@5@5@+=feaafiart1ev1aaatCvAUfKttLearuWrP9MDH5MBPbIqV92AaeXatLxBI9gBaebbnrfifHhDYfgasaacH8akY=wiFfYdH8Gipec8Eeeu0xXdbba9frFj0=OqFfea0dXdd9vqai=hGuQ8kuc9pgc9s8qqaq=dirpe0xb9q8qiLsFr0=vr0=vr0dc8meaabaqaciaacaGaaeqabaqabeGadaaakeaacqWGMbGzdaqhaaWcbaGaem4AaSMaeiikaGIaemyAaKMaeiykaKcabaGaeiikaGIaemyAaKMaeiykaKcaaOGaeyicI4SaemOray0aaSbaaSqaaiabdMgaPbqabaaaaa@39D1@ and N is the number of possible realizations. Given the values of all genes in network at time point *t *and a realization *f*_*k*_, the state of the genes after one updating step is expressed as

(*x*_1_(*t *+1), *x*_2_(*t *+1),... *x*_*n*_(*t *+1)) = *f*_*k*_(*x*_1_(*t*), *x*_2_(*t*),... *x*_*n*_(*t*))     (2)

Let f = (*f*^(1)^, *f*^(2)^,... *f*^(*n*)^) denote a random vector taking values in *F*_1 _× *F*_2 _⋯ × *F*_*n*_. The probability that a specific transition function fj(i)
 MathType@MTEF@5@5@+=feaafiart1ev1aaatCvAUfKttLearuWrP9MDH5MBPbIqV92AaeXatLxBI9gBaebbnrfifHhDYfgasaacH8akY=wiFfYdH8Gipec8Eeeu0xXdbba9frFj0=OqFfea0dXdd9vqai=hGuQ8kuc9pgc9s8qqaq=dirpe0xb9q8qiLsFr0=vr0=vr0dc8meaabaqaciaacaGaaeqabaqabeGadaaakeaacqWGMbGzdaqhaaWcbaGaemOAaOgabaGaeiikaGIaemyAaKMaeiykaKcaaaaa@3298@, (1 ≤ *j *≤ *l*(*i*)) is used to update gene *i *is equal to

cj(i)=Pr⁡{f(i)=fj(i)}=∑k:fk(i)(i)=fj(i)Pr⁡{f=fk}     (3)
 MathType@MTEF@5@5@+=feaafiart1ev1aaatCvAUfKttLearuWrP9MDH5MBPbIqV92AaeXatLxBI9gBaebbnrfifHhDYfgasaacH8akY=wiFfYdH8Gipec8Eeeu0xXdbba9frFj0=OqFfea0dXdd9vqai=hGuQ8kuc9pgc9s8qqaq=dirpe0xb9q8qiLsFr0=vr0=vr0dc8meaabaqaciaacaGaaeqabaqabeGadaaakeaacqWGJbWydaqhaaWcbaGaemOAaOgabaGaeiikaGIaemyAaKMaeiykaKcaaOGaeyypa0JagiiuaaLaeiOCaiNaei4EaSNaemOzay2aaWbaaSqabeaacqGGOaakcqWGPbqAcqGGPaqkaaGccqGH9aqpcqWGMbGzdaqhaaWcbaGaemOAaOgabaGaeiikaGIaemyAaKMaeiykaKcaaOGaeiyFa0Naeyypa0ZaaabuaeaacyGGqbaucqGGYbGCaSqaaiabdUgaRjabcQda6iabdAgaMnaaDaaameaacqWGRbWAcqGGOaakcqWGPbqAcqGGPaqkaeaacqGGOaakcqWGPbqAcqGGPaqkaaWccqGH9aqpcqWGMbGzdaqhaaadbaGaemOAaOgabaGaeiikaGIaemyAaKMaeiykaKcaaaWcbeqdcqGHris5aOGaei4EaShcbaGae8NzayMaeyypa0JaemOzay2aaSbaaSqaaiabdUgaRbqabaGccqGG9bqFaaa@6540@

**Figure 2:**
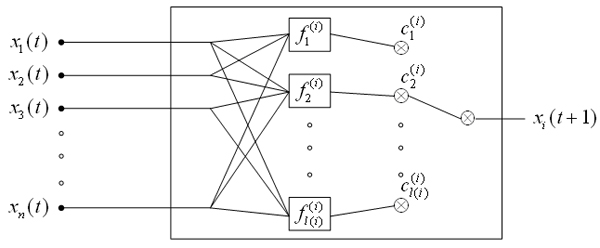
A basic building block of a PBN.

Given genes *V *= {*x*_1_, *x*_2_,..., *x*_*n*_}, each *x*_*i *_is assigned to a set of Boolean functions Fi={fj(i)}j=1,2,...l(i)
 MathType@MTEF@5@5@+=feaafiart1ev1aaatCvAUfKttLearuWrP9MDH5MBPbIqV92AaeXatLxBI9gBamXvP5wqSXMqHnxAJn0BKvguHDwzZbqegyvzYrwyUfgarqqtubsr4rNCHbGeaGqiA8vkIkVAFgIELiFeLkFeLk=iY=Hhbbf9v8qqaqFr0xc9pk0xbba9q8WqFfeaY=biLkVcLq=JHqVepeea0=as0db9vqpepesP0xe9Fve9Fve9GapdbaqaaeGacaGaaiaabeqaamqadiabaaGcbaGaemOray0aaSbaaSqaaiabdMgaPbqabaGccqGH9aqpcqGG7bWEcqWGMbGzdaqhaaWcbaGaemOAaOgabaGaeiikaGIaemyAaKMaeiykaKcaaOGaeiyFa03aaSbaaSqaaiabdQgaQjabg2da9iabigdaXiabcYcaSiabikdaYiabcYcaSiabc6caUiabc6caUiabc6caUiabdYgaSjabcIcaOiabdMgaPjabcMcaPaqabaaaaa@56D8@ to update target gene. The PBN will reduce to a standard Boolean network if *l*(*i*) = 1 for all genes. A basic building block of a PBN describing the updating mechanism is shown in Figure 2.

#### Construction of GRNs from PBN

The Coefficient of Determination (COD) is used to select a list of predictors for a given gene [19,23]. So far, most learning methods for reconstructing gene regulatory network use COD to select predictors for each target gene at any time point *t*. COD has also been used previously for the steady state data sets. Here we use upper case letters to represent random variables: Let *X*_*i *_be the target gene, X1(i),X2(i),…,Xl(i)(i)
 MathType@MTEF@5@5@+=feaafiart1ev1aaatCvAUfKttLearuWrP9MDH5MBPbIqV92AaeXatLxBI9gBaebbnrfifHhDYfgasaacH8akY=wiFfYdH8Gipec8Eeeu0xXdbba9frFj0=OqFfea0dXdd9vqai=hGuQ8kuc9pgc9s8qqaq=dirpe0xb9q8qiLsFr0=vr0=vr0dc8meaabaqaciaacaGaaeqabaqabeGadaaakeaacqWGybawdaqhaaWcbaGaeGymaedabaGaeiikaGIaemyAaKMaeiykaKcaaOGaeiilaWIaemiwaG1aa0baaSqaaiabikdaYaqaaiabcIcaOiabdMgaPjabcMcaPaaakiabcYcaSiablAciljabcYcaSiabdIfaynaaDaaaleaacqWGSbaBcqGGOaakcqWGPbqAcqGGPaqkaeaacqGGOaakcqWGPbqAcqGGPaqkaaaaaa@442B@ be sets of genes and f1(i),f2(i),…,fl(i)(i)
 MathType@MTEF@5@5@+=feaafiart1ev1aaatCvAUfKttLearuWrP9MDH5MBPbIqV92AaeXatLxBI9gBaebbnrfifHhDYfgasaacH8akY=wiFfYdH8Gipec8Eeeu0xXdbba9frFj0=OqFfea0dXdd9vqai=hGuQ8kuc9pgc9s8qqaq=dirpe0xb9q8qiLsFr0=vr0=vr0dc8meaabaqaciaacaGaaeqabaqabeGadaaakeaacqWGMbGzdaqhaaWcbaGaeGymaedabaGaeiikaGIaemyAaKMaeiykaKcaaOGaeiilaWIaemOzay2aa0baaSqaaiabikdaYaqaaiabcIcaOiabdMgaPjabcMcaPaaakiabcYcaSiablAciljabcYcaSiabdAgaMnaaDaaaleaacqWGSbaBcqGGOaakcqWGPbqAcqGGPaqkaeaacqGGOaakcqWGPbqAcqGGPaqkaaaaaa@447F@ be available Boolean functions. Thus, the optimal predictors of *X*_*i *_can be defined by f1(i)(X1(i)),f2(i)(X2(i)),…,fl(i)(i)(Xl(i)(i))
 MathType@MTEF@5@5@+=feaafiart1ev1aaatCvAUfKttLearuWrP9MDH5MBPbIqV92AaeXatLxBI9gBaebbnrfifHhDYfgasaacH8akY=wiFfYdH8Gipec8Eeeu0xXdbba9frFj0=OqFfea0dXdd9vqai=hGuQ8kuc9pgc9s8qqaq=dirpe0xb9q8qiLsFr0=vr0=vr0dc8meaabaqaciaacaGaaeqabaqabeGadaaakeaacqWGMbGzdaqhaaWcbaGaeGymaedabaGaeiikaGIaemyAaKMaeiykaKcaaOGaeiikaGIaemiwaG1aa0baaSqaaiabigdaXaqaaiabcIcaOiabdMgaPjabcMcaPaaakiabcMcaPiabcYcaSiabdAgaMnaaDaaaleaacqaIYaGmaeaacqGGOaakcqWGPbqAcqGGPaqkaaGccqGGOaakcqWGybawdaqhaaWcbaGaeGOmaidabaGaeiikaGIaemyAaKMaeiykaKcaaOGaeiykaKIaeiilaWIaeSOjGSKaeiilaWIaemOzay2aa0baaSqaaiabdYgaSjabcIcaOiabdMgaPjabcMcaPaqaaiabcIcaOiabdMgaPjabcMcaPaaakiabcIcaOiabdIfaynaaDaaaleaacqWGSbaBcqGGOaakcqWGPbqAcqGGPaqkaeaacqGGOaakcqWGPbqAcqGGPaqkaaGccqGGPaqkaaa@5D66@ and the probabilistic error measure can be represented as ε(Xi,fk(i)(Xk(i)))
 MathType@MTEF@5@5@+=feaafiart1ev1aaatCvAUfKttLearuWrP9MDH5MBPbIqV92AaeXatLxBI9gBaebbnrfifHhDYfgasaacH8akY=wiFfYdH8Gipec8Eeeu0xXdbba9frFj0=OqFfea0dXdd9vqai=hGuQ8kuc9pgc9s8qqaq=dirpe0xb9q8qiLsFr0=vr0=vr0dc8meaabaqaciaacaGaaeqabaqabeGadaaakeaaiiGacqWF1oqzcqGGOaakcqWGybawdaWgaaWcbaGaemyAaKgabeaakiabcYcaSiabdAgaMnaaDaaaleaacqWGRbWAaeaacqGGOaakcqWGPbqAcqGGPaqkaaGccqGGOaakcqWGybawdaqhaaWcbaGaem4AaSgabaGaeiikaGIaemyAaKMaeiykaKcaaOGaeiykaKIaeiykaKcaaa@413C@. For each k, the COD for *X*_*i *_relative to the conditioning set Xk(i)
 MathType@MTEF@5@5@+=feaafiart1ev1aaatCvAUfKttLearuWrP9MDH5MBPbIqV92AaeXatLxBI9gBaebbnrfifHhDYfgasaacH8akY=wiFfYdH8Gipec8Eeeu0xXdbba9frFj0=OqFfea0dXdd9vqai=hGuQ8kuc9pgc9s8qqaq=dirpe0xb9q8qiLsFr0=vr0=vr0dc8meaabaqaciaacaGaaeqabaqabeGadaaakeaacqWGybawdaqhaaWcbaGaem4AaSgabaGaeiikaGIaemyAaKMaeiykaKcaaaaa@327E@ is defined by

ωki=εi−ε(Xi,fk(i)(Xk(i)))εi     (4)
 MathType@MTEF@5@5@+=feaafiart1ev1aaatCvAUfKttLearuWrP9MDH5MBPbIqV92AaeXatLxBI9gBaebbnrfifHhDYfgasaacH8akY=wiFfYdH8Gipec8Eeeu0xXdbba9frFj0=OqFfea0dXdd9vqai=hGuQ8kuc9pgc9s8qqaq=dirpe0xb9q8qiLsFr0=vr0=vr0dc8meaabaqaciaacaGaaeqabaqabeGadaaakeaaiiGacqWFjpWDdaqhaaWcbaGaem4AaSgabaGaemyAaKgaaOGaeyypa0ZaaSaaaeaacqWF1oqzdaWgaaWcbaGaemyAaKgabeaakiabgkHiTiab=v7aLjabcIcaOiabdIfaynaaBaaaleaacqWGPbqAaeqaaOGaeiilaWIaemOzay2aa0baaSqaaiabdUgaRbqaaiabcIcaOiabdMgaPjabcMcaPaaakiabcIcaOiabdIfaynaaDaaaleaacqWGRbWAaeaacqGGOaakcqWGPbqAcqGGPaqkaaGccqGGPaqkcqGGPaqkaeaacqWF1oqzdaWgaaWcbaGaemyAaKgabeaaaaaaaa@4E54@

where *ε*_*i *_is the error of the best estimate of *X*_*i *_[23].

Now, if a class of gene sets X1(i),X2(i),…,Xl(i)(i)
 MathType@MTEF@5@5@+=feaafiart1ev1aaatCvAUfKttLearuWrP9MDH5MBPbIqV92AaeXatLxBI9gBaebbnrfifHhDYfgasaacH8akY=wiFfYdH8Gipec8Eeeu0xXdbba9frFj0=OqFfea0dXdd9vqai=hGuQ8kuc9pgc9s8qqaq=dirpe0xb9q8qiLsFr0=vr0=vr0dc8meaabaqaciaacaGaaeqabaqabeGadaaakeaacqWGybawdaqhaaWcbaGaeGymaedabaGaeiikaGIaemyAaKMaeiykaKcaaOGaeiilaWIaemiwaG1aa0baaSqaaiabikdaYaqaaiabcIcaOiabdMgaPjabcMcaPaaakiabcYcaSiablAciljabcYcaSiabdIfaynaaDaaaleaacqWGSbaBcqGGOaakcqWGPbqAcqGGPaqkaeaacqGGOaakcqWGPbqAcqGGPaqkaaaaaa@442B@ which have high CODs has been selected, we can use the optimal Boolean functions f1(i),f2(i),…,fl(i)(i)
 MathType@MTEF@5@5@+=feaafiart1ev1aaatCvAUfKttLearuWrP9MDH5MBPbIqV92AaeXatLxBI9gBaebbnrfifHhDYfgasaacH8akY=wiFfYdH8Gipec8Eeeu0xXdbba9frFj0=OqFfea0dXdd9vqai=hGuQ8kuc9pgc9s8qqaq=dirpe0xb9q8qiLsFr0=vr0=vr0dc8meaabaqaciaacaGaaeqabaqabeGadaaakeaacqWGMbGzdaqhaaWcbaGaeGymaedabaGaeiikaGIaemyAaKMaeiykaKcaaOGaeiilaWIaemOzay2aa0baaSqaaiabikdaYaqaaiabcIcaOiabdMgaPjabcMcaPaaakiabcYcaSiablAciljabcYcaSiabdAgaMnaaDaaaleaacqWGSbaBcqGGOaakcqWGPbqAcqGGPaqkaeaacqGGOaakcqWGPbqAcqGGPaqkaaaaaa@447F@ as the rule set for gene *X*_*i*_, with the probability of fj(i)
 MathType@MTEF@5@5@+=feaafiart1ev1aaatCvAUfKttLearuWrP9MDH5MBPbIqV92AaeXatLxBI9gBaebbnrfifHhDYfgasaacH8akY=wiFfYdH8Gipec8Eeeu0xXdbba9frFj0=OqFfea0dXdd9vqai=hGuQ8kuc9pgc9s8qqaq=dirpe0xb9q8qiLsFr0=vr0=vr0dc8meaabaqaciaacaGaaeqabaqabeGadaaakeaacqWGMbGzdaqhaaWcbaGaemOAaOgabaGaeiikaGIaemyAaKMaeiykaKcaaaaa@3298@ being chosen (see(3)). Then the approximations are given by

ck(i)=ωki∑j=1l(i)ωji     (5)
 MathType@MTEF@5@5@+=feaafiart1ev1aaatCvAUfKttLearuWrP9MDH5MBPbIqV92AaeXatLxBI9gBaebbnrfifHhDYfgasaacH8akY=wiFfYdH8Gipec8Eeeu0xXdbba9frFj0=OqFfea0dXdd9vqai=hGuQ8kuc9pgc9s8qqaq=dirpe0xb9q8qiLsFr0=vr0=vr0dc8meaabaqaciaacaGaaeqabaqabeGadaaakeaacqWGJbWydaqhaaWcbaGaem4AaSgabaGaeiikaGIaemyAaKMaeiykaKcaaOGaeyypa0ZaaSaaaeaaiiGacqWFjpWDdaqhaaWcbaGaem4AaSgabaGaemyAaKgaaaGcbaWaaabmaeaacqWFjpWDdaqhaaWcbaGaemOAaOgabaGaemyAaKgaaaqaaiabdQgaQjabg2da9iabigdaXaqaaiabdYgaSjabcIcaOiabdMgaPjabcMcaPaqdcqGHris5aaaaaaa@46DE@

According to the above expressions [19,23], those Boolean functions corresponding to the highest CODs will be selected in the probabilistic network. The selected Boolean functions are used to predict the gene expression status at the next time point, and they also will be used to reconstruct gene regulatory networks.

### Bayesian networks and dynamic Bayesian networks

Among the many computational approaches that infer gene regulatory networks from time series data, Bayesian network analysis draws significant attention because of its probabilistic nature. DBN is the temporal extension of Bayesian network analysis. It is a general model class that is capable of representing complex temporal stochastic processes. It captures several other often used modeling frameworks as its special cases, such as hidden Markov models (and its variants) and Kalman filter models.

#### Bayesian network

Given a set of variables *U *= {*x*_1_, *x*_2_,... *x*_*n*_} in gene network, a Bayesian network, for *U *is a pair *B *= (*G*, Θ) which encodes a joint probability distribution over all states of *U*. It is composed of a directed acyclic graph (DAG) *G *whose nodes correspond to the variables in *U *and Θ which defines a set of local conditional probability distributions (CPD) to qualify the network. Let Pa(*x*_*i*_) denote the parents of the variables *x*_*i *_in the acyclic graph *G *and pa(*x*_*i*_) denote the values of the corresponding variables. Given *G *and Θ, a Bayesian network defines a unique joint probability distribution over U given by

Pr⁡{x1,x2,...xn}=∏i=1nPr⁡{xi|pa(xi)}     (6)
 MathType@MTEF@5@5@+=feaafiart1ev1aaatCvAUfKttLearuWrP9MDH5MBPbIqV92AaeXatLxBI9gBaebbnrfifHhDYfgasaacH8akY=wiFfYdH8Gipec8Eeeu0xXdbba9frFj0=OqFfea0dXdd9vqai=hGuQ8kuc9pgc9s8qqaq=dirpe0xb9q8qiLsFr0=vr0=vr0dc8meaabaqaciaacaGaaeqabaqabeGadaaakeaacyGGqbaucqGGYbGCcqGG7bWEcWaJaoiEaG3aiWiGBaaaleacmcOamWiGigdaXaqajWiGaOGaeiilaWIamWiGdIha4nacmc4gaaWcbGaJakadmciIYaGmaeqcmciakiabcYcaSiabc6caUiabc6caUiabc6caUiadmc4G4baEdGaJaUbaaSqaiWiGcWaJaoOBa4gabKaJacGccqGG9bqFcqGH9aqpdaqeWbqaaiGbccfaqjabckhaYjabcUha7jabdIha4naaBaaaleaacqWGPbqAaeqaaOGaeiiFaWhcbaGae8hCaaNae8xyaeMaeiikaGIaemiEaG3aaSbaaSqaaiabdMgaPbqabaGccqGGPaqkcqGG9bqFaSqaaiabdMgaPjabg2da9iabigdaXaqaaiab=5gaUbqdcqGHpis1aaaa@68A8@

For more detail on Bayesian networks, see [24].

#### Dynamic Bayesian network

A DBN is defined by a pair (*B*_0_, *B*_1_) represents the joint probability distribution over all possible time series of variables X = {*X*_1_, *X*_2_,... *X*_*n*_}, where *X*_*i*_(1 ≤ *i *≤ *n*) represents the binary-valued random variables in the network, besides, we use lower case *x*_*i *_(1 ≤ *i *≤ *n*) denotes the values of variable *X*_*i*_. It is composed of an initial state of Bayesian network *B*_0 _= (*G*_0_, Θ_0_) and a transition Bayesian network *B*_1 _= (*G*_1_, Θ_1_), where *B*_0_specifies the joint distribution of the variables in *X*(0) and *B*_1 _represents the transition probabilities Pr{X(*t *+ 1) | X(*t*)} for all *t*. In slice 0, the parents of *X*_*i*_(0) are assumed to be those specified in the prior network *B*_0_, which means Pa(*X*_*i*_(0)) ⊆ X(0) for all 1 ≤ *i *≤ *n*; in slice *t *+ 1, the parents of *X*_*i*_(*t *+ 1) are nodes in slices *t*, Pa(*X*_*i*_(*t *+ 1)) ⊆ X(*t*) for all 1 ≤ *i *≤ *n *and *t *≥ 0, as stated in [25], the connections only exist between consecutive slices. The joint distribution over a finite list of random variables X(0) ∪ X(1) ∪ ⋯ ∪ X(T) can be expressed as [24,25]

Pr⁡{x(0),x(1),...,x(T)}=Pr⁡{x(0)}∏t=0T−1Pr⁡{x(t+1)|x(t)}=∏i=1nPr⁡{xi(0)|pa(Xi(0))}×∏t=0T−1∏j=1nPr⁡{xj(t+1)|pa(Xj(t+1))}     (7)
 MathType@MTEF@5@5@+=feaafiart1ev1aaatCvAUfKttLearuWrP9MDH5MBPbIqV92AaeXatLxBI9gBaebbnrfifHhDYfgasaacH8akY=wiFfYdH8Gipec8Eeeu0xXdbba9frFj0=OqFfea0dXdd9vqai=hGuQ8kuc9pgc9s8qqaq=dirpe0xb9q8qiLsFr0=vr0=vr0dc8meaabaqaciaacaGaaeqabaqabeGadaaakeaafaqaaeWabaaabaGagiiuaaLaeiOCaiNaei4EaShcbaGae8hEaGNaeiikaGIaeGimaaJaeiykaKIaeiilaWIae8hEaGNaeiikaGIaeGymaeJaeiykaKIaeiilaWIaeiOla4IaeiOla4IaeiOla4IaeiilaWIae8hEaGNaeiikaGIae8hvaqLaeiykaKIaeiyFa0habaGaeyypa0JagiiuaaLaeiOCaiNaei4EaSNae8hEaGNaeiikaGIaeGimaaJaeiykaKIaeiyFa03aaebCaeaacyGGqbaucqGGYbGCcqGG7bWEcqWF4baEcqGGOaakcqWG0baDcqGHRaWkcqaIXaqmcqGGPaqkcqGG8baFcqWF4baEcqGGOaakcqWG0baDcqGGPaqkaSqaaiab=rha0jabg2da9iabicdaWaqaaiab=rfaujabgkHiTiabigdaXaqdcqGHpis1aOGaeiyFa0habaGaeyypa0ZaaebCaeaacyGGqbaucqGGYbGCcqGG7bWEcqWG4baEdaWgaaWcbaGaemyAaKgabeaakiabcIcaOiabicdaWiabcMcaPiabcYha8jab=bhaWjab=fgaHjabcIcaOiabdIfaynaaBaaaleaacqWGPbqAaeqaaOGaeiikaGIaeGimaaJaeiykaKIaeiykaKIaeiyFa0Naey41aq7aaebCaeaadaqeWbqaaiGbccfaqjabckhaYjabcUha7jabdIha4naaBaaaleaacqWGQbGAaeqaaOGaeiikaGIaemiDaqNaey4kaSIaeGymaeJaeiykaKIaeiiFaWNae8hCaaNae8xyaeMaeiikaGIaemiwaG1aaSbaaSqaaiabdQgaQbqabaGccqGGOaakcqWG0baDcqGHRaWkcqaIXaqmcqGGPaqkcqGGPaqkcqGG9bqFaSqaaiabdQgaQjabg2da9iabigdaXaqaaiabd6gaUbqdcqGHpis1aaWcbaGaemiDaqNaeyypa0JaeGimaadabaGae8hvaqLaeyOeI0IaeGymaedaniabg+GivdaaleaacqWGPbqAcqGH9aqpcqaIXaqmaeaacqWGUbGBa0Gaey4dIunaaaaaaa@B4DF@

**Figure 3:**
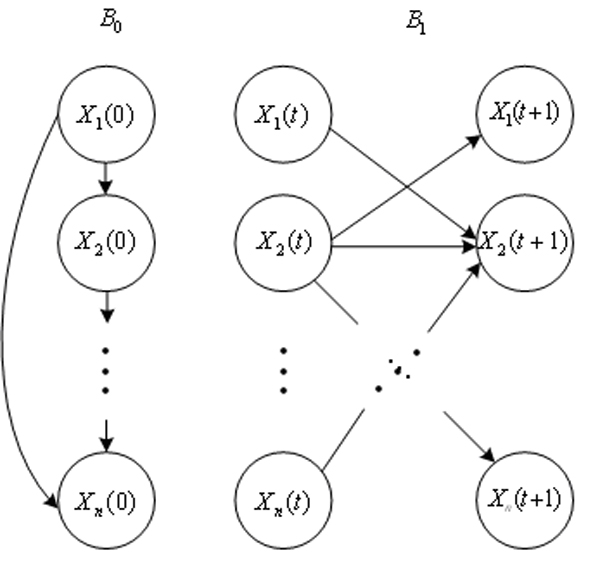
A basic building block of a DBN.

An example of a DBN is shown in Figure 3.

#### Construction of GRNs from DBN

Given a set of training gene data, how the network structure is found that best fits the data is called learning the structure of a dynamic Bayesian network. The goal of constructing a network is to find the model with maximum likelihood (i.e., REVEAL algorithm in [3] and its improvement in [9]). The network we want to learn is the transition network, i.e., the network defining dependencies between the adjacent time slices *X*(*t*) and *X*(*t *+ 1). The training set of data is composed of all adjacent time-slices *X*(*t*) and *X*(*t *+ 1).

Algorithms for learning gene network structure have focused on networks with complete data. Structural Expectation Maximization (SEM) is developed to handle data with hidden variables and missing values. One of the algorithms to infer network structure from training data is based on the mutual information analysis of the data. For each node, this algorithm learns the optimal parent set independently by choosing the parent set that maximizes a scoring function. The scoring function is defined by

I(*X*, Pa(*X*))/max{*H*(*X*), *H*(Pa(*X*))}     (8)

where *I*(*X*, *Y*) is the mutual information between *X *and *Y*, and *H*(*X*) is the entropy of *X*. With parent set of genes in DBN, GRNs can be constructed [31].

For each inferred network, scoring metrics are used to evaluate the probabilistic scores which explain relationships in the given data sets. There are two popular Bayesian scoring metrics: the BDe (Bayesian Dirichlet equivalence) score [32] and the BIC (Bayesian information criterion) score [33]. Then, the network with highest score will be identified using search heuristics, which have three widely used methods: greedy search, simulated appealing and a genetic algorithm [10].

## Competing interests

The authors declare that they have no competing interests.

## Authors' contributions

PL implemented the algorithms and inferred gene networks. PL and CZ performed the statistical analysis and drafted the manuscript. CZ and YD coordinated the study. EP, PG and YD gave suggestions to improve the methods and revised the manuscript. All authors read and approved the final manuscript.
